# Poisonous Soups

**Published:** 1869-01

**Authors:** 


### POISONOUS SOUPS.

"Extract of meat" prepared by Prof. Liebig's method is pronounced poisonous by Dr. Kemmerich, and many Journals have taken up the cry of mad dog, and advise the public to use no more of this preparation for soups, to be used by invalids and others, on the ground that it abounds in potash-salts. The effect of large doses, though small doses may be useful as a tonic, is to destroy life by paralysis of the heart, as shown by an experiment on a dog. The utter absurdity of such a statement ought to have prevented its being copied into the journals of the country, at least until some of our standard medical journals had pronounced upon the subject.

There is scarcely an article of food or a condiment which some ignoramus has not pronounced prejudicial to life if used, although men have been using them since the world began. Whole chapters have been written in right down earnest, that common salt as used in our food by the masses, is slowly destroying the constitutions of men. Within a few months the papers have been teeming with objurgations against the use of pork in any form, because its tendency was to fill the human body with worms which multiplied at the rate of a million a minute or thereabouts; and one would have thought that if pork was not banished from our tables on the spot, all the people would have been killed by wormy convulsions before Christmas. Persons write to us to know if they had not better throw all their snow-white hogs' lard into the streets or sink-holes.

Then came the cry that our domestic divinities, in the use of the hair of others for the adornment of their heads, were becoming infected with a terrible parasite which adhered to "false" hair, and a long unpronounceable German name was given as authority sufficient to banish the use of false hair from every woman's head in the nation, and yet pork and hogs' lard, and table salt, and false hair are used as much as before, and still the human race has not perished from the face of the earth by using salt, nor gone into convulsions by eating "ham and eggs," nor become wormy by wearing waterfalls.

Now, this new terror is to become a nine days' wonder, that a simple preparation of beef-steak is fatally poisonous, because it has the elements of potash, and pure potash will kill a dog. Roast beef will kill a man if too much of it has been eaten; we have read within twenty-four hours, that a man just married had killed himself by drinking too much cold water. Many an unfortunate has died of an overdose of love; of too much joy. That Liebig's extract of beef may have poisoned some persons is not unlikely, but it was because it was improperly prepared or put up improperly, so as to corrode the vessels which contained it. If properly prepared extract of beef is put up in incorrosive vessels, say of wood or glass, it is utterly impossible for it to possess one atom of a poisonous quality other than glass-canned fruit, or tomatoes, or vegetables contain.

